# Investigating intra‐tumoural heterogeneity and microenvironment diversity in primary cardiac angiosarcoma through single‐cell RNA sequencing

**DOI:** 10.1002/ctm2.70113

**Published:** 2024-12-10

**Authors:** Jingyuan Huo, Zhen Wang, Wenting Zhao, Miao Chen, Haoyang Li, Fengpu He, Xiao Tian, Yaqi Ma, Firyuza Husanova, Liang Ma, Yiming Ni, Hongda Ding, Weidong Li, Hongfei Xu

**Affiliations:** ^1^ Department of Cardiovascular Surgery School of Medicine the First Affiliated Hospital of Zhejiang University Hangzhou China; ^2^ Department of Cardiology School of Medicine the First Affiliated Hospital of Zhejiang University Hangzhou China; ^3^ Department of Pathology School of Medicine the First Affiliated Hospital of Zhejiang University Hangzhou China; ^4^ Department of General Surgery Shengjing Hospital of China Medical University Shenyang China

**Keywords:** heterogeneity, multicolour immunohistochemistry, primary cardiac angiosarcoma, single‐cell RNA sequencing, tumour microenvironment

## Abstract

**Background:**

Primary cardiac angiosarcoma (PCAS) is a rare and aggressive heart tumour with limited treatment options and a poor prognosis. Understanding cellular heterogeneity and tumour microenvironment (TME) is crucial for the development of effective therapies. Here, we investigated the intratumoural heterogeneity and TME diversity of PCAS using single‐cell RNA sequencing (scRNA‐seq).

**Methods:**

We performed scRNA‐seq analysis on tumour samples from four patients with PCAS, supplemented with multicolour immunohistochemistry for identification. We used scRNA‐seq data from five normal cardiac tissue samples downloaded from public databases for comparative analyses. Bioinformatic analyses, including Cell Ranger, Seurat, Monocle2, hdWGCNA, SCENIC and NicheNet, were utilized to identify distinct cell populations, transcriptional patterns, and co‐regulating gene modules.

**Results:**

Our analysis revealed significant intratumoural heterogeneity in PCAS driven by diverse biological processes such as protein synthesis, degradation, and RIG‐I signalling inhibition. The SCENIC analysis identified three primary transcription factors' clusters (*CEBPB*, *MYC* and *TAL1*). T‐cell subset analysis showed exhausted antigen‐specific T‐cells, complicating the efficacy of immune checkpoint blockade. Furthermore, we observed suppressive macrophages (SPP1+ and OLR1+) and reduced mitochondrial gene *MT‐RNR2* (MTRNR2L12) expression in TME‐infiltrating cells, indicating impaired mitochondrial function.

**Conclusion:**

This study elucidates the complex cellular landscape and immune microenvironment of PCAS, highlighting potential molecular targets for the development of novel therapies. These findings underscore the importance of a multifaceted therapeutic approach for addressing the challenges posed by PCAS's heterogeneity and immune evasion.

**Key points:**

- Insights into the heterogeneity and transcriptional patterns of sarcoma cells may explain the challenges in treating primary cardiac angiosarcoma (PCAS) using the current therapeutic modalities.
- Characterization of the immune microenvironment revealed significant immunosuppression mediated by specific myeloid cell populations (SPP1+ and OLR1+ macrophages).
- Identification of mitochondrial dysfunction in immune cells within the PCAS microenvironment, particularly the notable downregulation of the MTRNR2L12 protein, suggests a new avenue for therapeutic targeting.

## Graphical abstract

- Insights into sarcoma cell heterogeneity and transcriptional patterns explain challenges in treating primary cardiac angiosarcoma (PCAS) with current therapies.
- Characterization of the immune microenvironment revealed significant immunosuppression mediated by specific myeloid cell populations (SPP1+ and OLR1+ macrophages).
- Mitochondrial dysfunction in immune cells, particularly the downregulation of MTRNR2L12, suggests a new therapeutic target in PCAS.

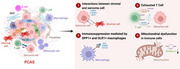

## 1 INTRODUCTION

Primary cardiac angiosarcoma (PCAS) is a rapidly progressing primary cardiac malignancy. It frequently manifests in the atrium, affecting individuals aged 20–50 years, where it leverages a dense vascular network. PCAS, accounting for ∼40% of cardiac sarcomas, is the most common and differentiated form of cardiac sarcoma.^1^ The prognosis is grim, with the median overall survival post‐diagnosis being 5–13 months. This poor outcome is attributed to the aggressive biological behaviour of the tumour, the complexity of achieving complete surgical resection, and its resistance to radiotherapy and chemotherapy.^2^, ^3^ PCAS treatment is limited by the scarcity of targeted therapies.^4^, ^5^ Further, it is rare and etiologically and genetically under‐characterized, hindering the development of effective treatment.

Prior transcriptomic sequencing and genome‐wide single nucleotide polymorphism analyses have revealed critical insights into PCAS–driver mutations, regulatory anomalies, and disease subtypes.^6^ Aberrations such as trisomies in chromosomes 4, 8, 11, 17, and 20, as well as gains in 1q+ and homozygous deletions of CDKN2 have been documented.^7^ Furthermore, somatic mutations in genes such as *KRAS*, *TP53* and *PLCG1* have been observed.^8^, ^9^ However, traditional bulk profiling methods fall short of distinguishing among cell types, thereby obscuring the nuances of intra‐ and inter‐tumour heterogeneity.

Single‐cell RNA sequencing (scRNA‐seq) overcomes these limitations by elucidating the gene expression profiles of individual cells, facilitating the identification of novel cellular subtypes.^10^, ^11^ It can finely map cell components and their distribution within the immune microenvironment,^12^, ^13^ analyze the impact of extrinsic factors on the tumour milieu,^14^ elucidate the evolutionary dynamics of immune cells within the microenvironment,^15^ decode the molecular underpinnings of the tumour's suppressive environment,^16^ and unearth new therapeutic targets alongside prognostic indicators.^17^ In this study, we aimed to meticulously examine PCAS characteristics and its tumour microenvironment (TME) using scRNA‐seq and multicolour immunohistochemistry (IHC).

## 2 MATERIALS AND METHODS

### 2.1 PCAS patient samples collection and resources of public data

Human PCAS specimens were obtained from The First Affiliated Hospital of Zhejiang University College of Medicine (Zhejiang, China). The diagnosis was established through transesophageal echocardiography (TEE), positron emission tomography/computed tomography (PET‐CT), morphological assessment, and immunohistochemical analysis of angiosarcoma markers, including MDM2, Ki67 (MKI67), CD34, and Fli‐1 (FLI1/rna_FLI1). Tumour debulking was performed using minimally invasive surgical techniques via right mini‐thoracotomy, during which tumour tissues were collected for analysis.

Over two years, ten fresh samples suspected of cardiac malignancy were subjected to scRNA‐seq. Out of these, four were confirmed as PCAS, and the remaining six were identified as other cardiac malignancies. Patients did not receive any treatment before the surgical procedures. Samples were promptly cleansed with cold phosphate‐buffered saline (PBS) to remove blood contamination. For scRNA‐seq, fresh tissue specimens were promptly transported to the laboratory in a specialized cell preservation solution.

All patients provided informed consent for the use of their tissues in this study, which was approved by the Clinical Research Ethics Committee of First Affiliated Hospital, Zhejiang University School of Medicine.

Public data were obtained from Litviňuková et al., *Cells of the adult human heart* (Nature, 2020), which includes single‐cell RNA sequencing from seven healthy left ventricular samples. Two samples were excluded due to low cell counts, leaving five samples with 16,764 cells in total (cell counts: 1151, 2215, 613, 6470 and 6315). Data are accessible at https://www.ebi.ac.uk/ena/browser/text‐search?query=ERP123138, and further details at https://doi.org/10.1038/s41586‐020‐2797‐4.

For quality control, we filtered and analyzed data with Seurat V4.0 and applied Harmony for batch‐effect correction. Parameters are provided in Table S1.

### 2.2 Tissue dissociation and single‐cell isolation

Single‐cell suspensions were generated from flash‐frozen tumour tissues treated with an RNase‐free lysis solution. The entire tumour mass was sectioned into small fragments using a sterilized razor blade, followed by immediate immersion in ice‐cold lysis buffer composed of 10 mM Tris‐HCl (pH 7.4), 10 mM NaCl, 3 mM MgCl_2_, 0.1% Tween‐20, 0.1% Nonidet P40 Substitute, 0.01% digitonin, and 1% bovine serum albumin (BSA) dissolved in nuclease‐free water. These tissue pieces were then subjected to enzymatic dissociation in a digestion medium containing dispase, DNase, and trypsin.

The digested tissue homogenate was gently pipetted using a wide‐bore pipette to facilitate cell separation. The resulting cell suspension was passed through a cell strainer to remove any undissolved fragments. To clean the cells of any remaining enzymes and detritus, they were rinsed with a PBS‐balanced salt solution. Cell quantification was conducted using a hemocytometer, which also assisted in adjusting the cell density to meet the requirements for subsequent processes. Finally, cells were resuspended at a suitable concentration in the selected medium or buffer to prepare them for scRNA‐seq analysis.

### 2.3 10x Library preparation and scRNA‐seq

For the transcriptomic profiling at the single‐cell level, we utilized the Single Cell 3′ Library & Gel Bead Kit V3 (10x Genomics, 1000075) and the Chromium Single Cell B Chip Kit (10x Genomics, 1000074). Overall, 50,782 cells from the tumour samples were loaded onto a Chromium Single Cell Controller (10x Genomics) to generate single‐cell gel beads‐in‐emulsion (GEMs), according to the manufacturer's guidelines. The cells were then resuspended in PBS containing 0.04% BSA. Approximately 6000 cells were allocated to each channel of the controller, aiming for a recovery target of roughly 3000 cells per channel. Once captured within the GEMs, the cells were lysed and the liberated RNA was barcoded during the reverse transcription process conducted within each GEM. This reverse transcription occurred in an S1000 Touch Thermal Cycler (Bio‐Rad) at 53°C for 45 min, followed by a 5‐min incubation at 85°C, and then the samples were held at 4°C. After cDNA generation and amplification, the quality of the libraries was evaluated using an Agilent 4200 instrument, a service provided by CapitalBio Technology, Beijing. The sequencing libraries prepared using the Single Cell 3′ Library & Gel Bead Kit V3, were sequenced on an Illumina NovaSeq 6000 system. This was done to achieve a minimum sequencing depth of 100,000 reads per cell employing a PE150 (pair‐end 150 bp) reading strategy, a procedure also carried out by CapitalBio Technology, Beijing.

### 2.4 scRNA‐seq data processing

The Cell Ranger software suite acquired from the 10x Genomics website was used for data processing. The ‘cell ranger count’ module facilitated alignment, filtering, barcode, and unique molecular identifier counting to generate a feature‐barcode matrix and delineate clusters. Dimensionality reduction was executed via principal component analysis, utilizing the leading ten principal components to construct clusters using both K‐means and graph‐based clustering algorithms. An alternative clustering method was applied using the Seurat 3.0 R package. During quality control, cells were excluded if they expressed fewer than 200 genes, were in the top 1% of gene expression, or had mitochondrial gene content exceeding 25%. Data visualization employs t‐distributed stochastic neighbour embedding (Uniform Manifold Approximation and Projection) techniques.

Functional enrichment analyses, including Gene Ontology, Kyoto Encyclopedia of Genes and Genomes (KEGG), Reactome, and disease‐related enrichment (human only), were conducted using KOBAS software. This analysis used the top 20 marker genes from each cluster, adjusted for multiple testing using the Benjamini–Hochberg procedure. The enrichment results were graphically rendered using the R package. Protein–protein interaction data were sourced from the STRING database, requiring a minimum combined score of 400. We extracted interactions for the top 20 marker genes from each cluster and illustrated the interaction networks using Cytoscape software.

### 2.5 Copy number variation analysis

The initial copy number variation (CNV) analysis was performed using the inferCNV R package (version 1.3.3). We used the R package (cutoff value = 0.1) to obtain the residual expression of each gene as a CNV substitute. The CNV score for each cell was calculated using a quadratic sum. To study the CNV level in each cell type for each sample, we used the average estimated CNV of the cells as a background to eliminate individual CNV.

### 2.6 Pseudotime trajectory analysis

Seurat identified the differentially expressed genes (DEGs) in malignant and epithelial cells. The Monocle2 package (v2.8.0) was used to perform trajectory analysis, creating pseudotemporal trajectories with the top 300 DEGs from both cell types. We traced the progression of DEGs over pseudotime, moving from normal epithelial cells with atypical gene expression to the malignant transformation, using Monocle2's “differentialGeneTest” function.

### 2.7 SCENIC analysis

We constructed a co‐expression network following the procedures outlined on the webpage https://www.nature.com/articles/s41596‐020‐0336‐2 (Nature Protocols, 2020).^18^ We then identified potential targets for each transcription factor (TF) based on their co‐expression profiles. The subsequent steps included the selection of potential direct‐binding targets through DNA motif analysis. We conducted TF enrichment analysis of gene sets to examine the activity of regulons in individual cells. Finally, we determined the stable cell states based on regulon activity and explored the implications of these findings.

### 2.8 High‐dimensional weighted gene co‐expression network analysis

We established an R conda environment for high‐dimensional weighted gene co‐expression network analysis (hdWGCNA). Subsequently, we launched R and installed the necessary dependencies including packages from Bioconductor, Seurat, WGCNA, igraph, and devtools. For hdWGCNA analysis, we followed the detailed steps provided in the protocol published on the website https://www.nature.com/articles/s41588‐021‐00894‐z (Nat Genet, 2021).^19^

### 2.9 NicheNet analysis

The original dataset was processed and aggregated using the Seurat alignment pipeline. After loading NicheNet's networks, we performed the NicheNet analysis.^20^ Initially, we defined a ‘receiver/target’ cell population and identified expressed genes. For the sender‐focused approach, we specified sender cell types and catalogue‐expressing genes in all sender populations. We then defined the gene set of interest along with the background genes. Using the top 30 ligands, we predicted active target genes and constructed an active ligand‐receptor network. We then identified the target genes and receptors of the top‐ranked ligands. The analysis culminated in sender‐focused evaluations and summary visualizations of the NicheNet findings.

### 2.10 Multicolor IHC staining

PACS tumour samples were used for IHC staining. Antibodies such as: anti‐CD31 (ab9498, 1:100), anti‐CEBPB (ab32358, 1:100), anti‐MYC (ab32072, 1:100), anti‐TAL1 (ab155195, 1:100) and anti‐CD3 (ab16669, 1:150), anti‐PDCD1 (ab52587, 1:50), anti‐STAT4 (ab284408, 1:500), anti‐CD11b (GB11058, 1:50), anti‐OLR1 (ab126538, 1:200) and anti‐SPP1 (ab214050, 1:1000) were used in this study. All staining processes were performed using an IHC/ISH System (BenchMark GX, Roche) following the manufacturer's instructions.

## 3 RESULTS

### 3.1 Identification of cell populations in the cardiac sarcoma's tissues

**FIGURE 1:**
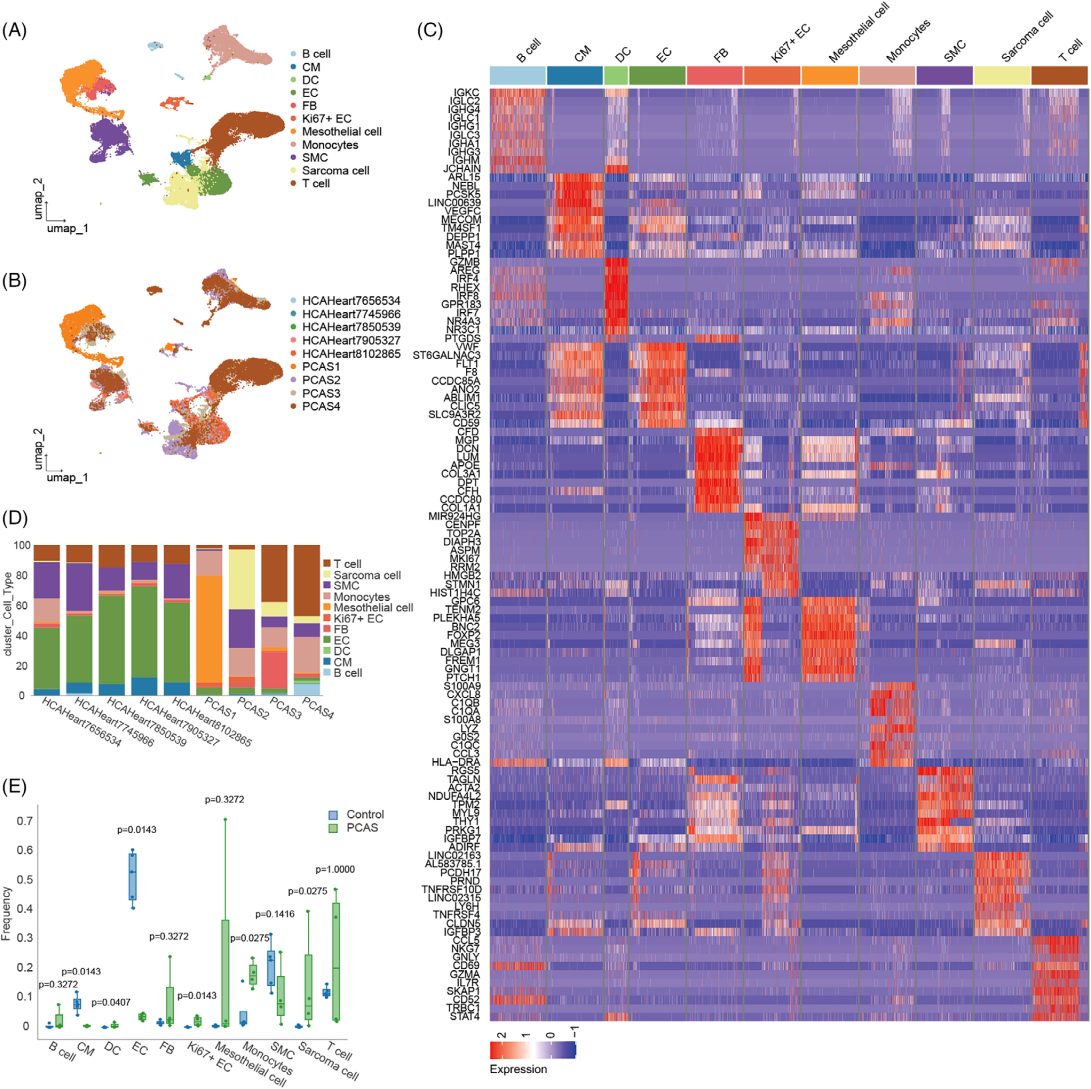
Landscape of cells in the sarcoma tissues and normal cardiac tissues from patients and volunteers. (A) Uniform manifold approximation and projection (UMAP) plot of 10x genomics‐based single cells showing 11 major cell types by manual annotation. (B) UMAP plot of 10x genomics‐based single cells showing nine samples by manual annotation. (C) Heatmap plot showing expression levels of selected marker genes for each cell type. (D) Cluster distribution of the 11 major cell types showing frequencies per sample. (E) Boxplot showing the proportion of major cell types from patients (*n* = 4) or volunteers (*n* = 5). Public data for the normal control group were sourced from Litviňuková et al., *Cells of the adult human heart* (Nature, 2020) and included single‐cell RNA sequencing data from seven healthy left ventricular tissues. After filtering for cell counts, five samples were retained with a total of 16,764 cells. *p‐*Value was determined by unpaired Student's *t*‐test.

The scRNA‐seq data were generated from cardiac sarcoma tissues of four patients, which were then integrated with existing scRNA‐seq datasets from normal cardiac tissues for comparative analysis.^21^ Figure S1 details the clinical profiles and diagnostic information of the cardiac sarcoma patients and Hematoxylin and Eosin stain results. After quality control, clustering, and marker gene identification, 41,346 single cells were analyzed, revealing 11 major cell populations comprising 22 distinct subsets (Figure 1A–C and Figure S2A). Notable differences in cell‐type composition were observed between the normal and PCAS groups (Figure 1D), marked by an increase in monocytes, T cells, and sarcoma cells, including mesothelial cells, and a decrease in endothelial cells (ECs, Figure S2B). Notably, mesothelial cells were predominant in one patient, underscoring the considerable heterogeneity among the four patient samples (Figure 1E). CellPhoneDB analysis highlighted extensive interactions between stromal and sarcoma cells,^22^ with monocytes being the primary immune cells exhibiting close communication with sarcoma cells (Figure S2C,D). Furthermore, we explored immune cell infiltration using the public angiosarcoma database (GSE163359), which was partly consistent with our analysis (Figure S2E).

### 3.2 Identification of cell states related to sarcomas

**FIGURE 2:**
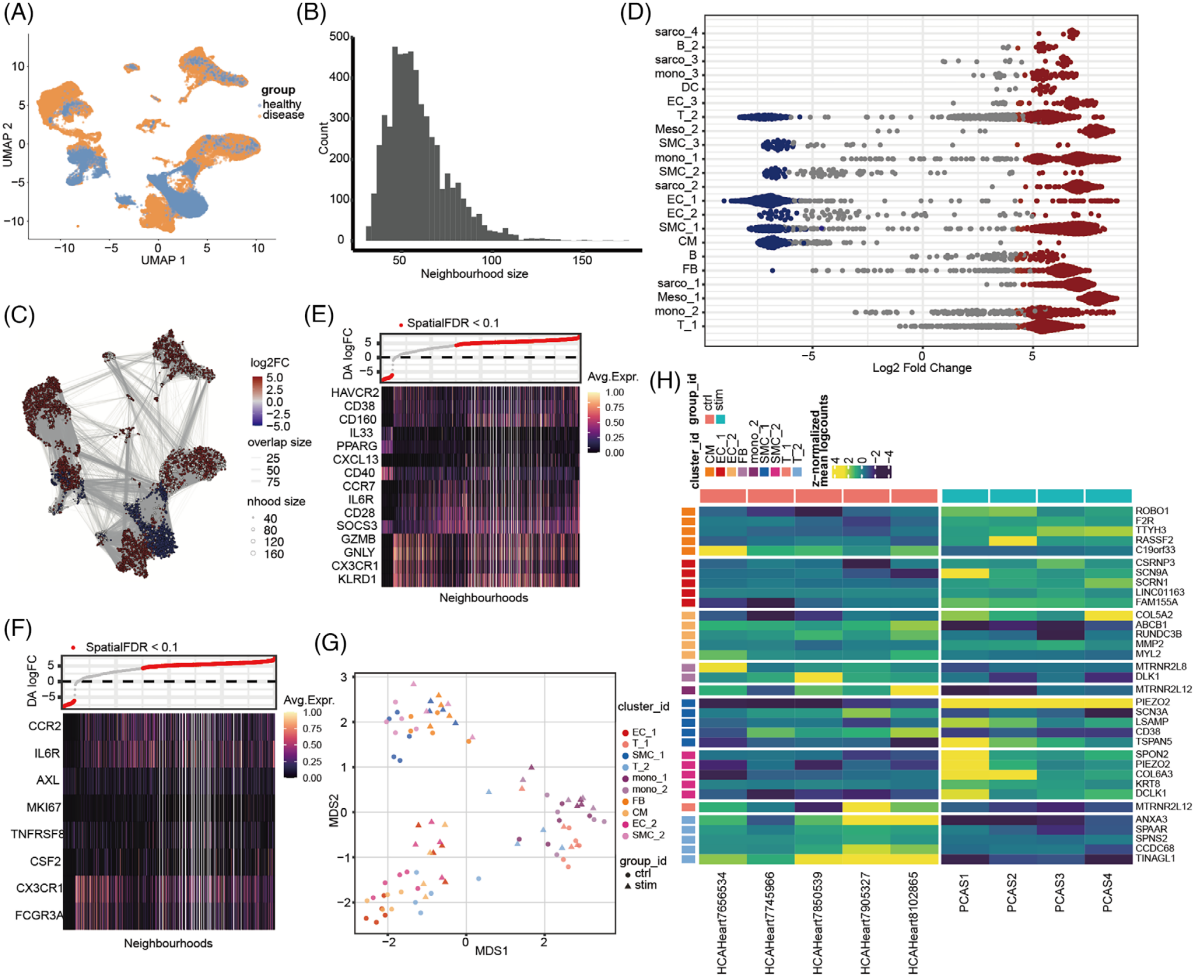
Landscape of T cells and myeloid cells. (A) Uniform manifold approximation and projection (UMAP) plot of 10x genomics‐based single cells showing two phenotypes by manual annotation. (B) Bar plot showing distributions of neighbourhood size. (C) Colours denote the assignment of neighbourhoods to discrete groups using Louvain clustering. The region encircled by the dashed line denotes neighbourhood groups that correspond to the disease phenotype. (D) Beeswarm plot of the distribution of log fold change across age in neighbourhoods containing cells from different cell type clusters. (E, F) Heatmap plots showing differentially expressed genes between differential abundance neighbourhoods in the inter‐typical T cells (E) or myeloid cells (F). Columns are neighborhoods and rows are differentially expressed genes (FDR 5%). The top panel denotes the neighbourhood log fold change. (G) Pseudobulk‐level multidimensional scaling (MDS) plot. Each point represents one subpopulation‐sample instance; points are coloured by subpopulation and shaped by group ID. (H) Heatmap of pseudobulk‐level log‐expression values normalized to the mean of vehicle samples; rows correspond to genes, columns to subpopulation‐sample combinations. Included is the union of DS detections (false discovery rate [FDR] < 0.05 and log_2_FC > 1) across all subpopulations.

Most immune cells such as monocytes, T cells, and sarcoma cells were predominantly found in the PCAS group. This prevalence enabled a direct comparison of differential infiltration between the groups. To delineate disease‐related cell state trajectories, we conducted a Milo analysis (Figure 2A).^23^ We initiated by constructing a K Nearest Neighbours graph (k = 20) and identified 682 cell neighbourhoods (Figure 2B). At a 10% false discovery rate (FDR), a differential abundance test highlighted 479 neighbourhoods with a significantly altered abundance (Figure 2C). Sarcoma cells, fibroblasts (FB), numerous T cells, and monocytes showed increased abundance associated with the disease, whereas smooth muscle cells (SMCs) and ECs were less abundant (Figure 2D). Furthermore, we explored the subtypes of T cells and monocytes within these neighbourhoods using marker gene expression profiling. T‐cells in the sarcoma microenvironment exhibited elevated levels of cytotoxicity (*GZMB*, *GNLY* and *KLRD1*), exhaustion (*HAVCR2*, *CD38*, *CD160*, *CXCL13* and *CX3CR1*), and inflammation (*IL33*, *PPARG*, *IL6R* and *SOCS3*), suggesting an activated but exhausted phenotype (Figure 2E). In contrast, monocytes showed increased chemotaxis (*CCR2* and *CX3CR1*) and proliferation (*MKI67* and *CSF2*), but not inflammation (Figure 2F).

To comprehensively assess differential gene expression in disease states, we utilized Muscat for cross‐condition differential state analysis based on pseudo bulk data aggregation (Figure 2G).^24^ Notably, *MTRNR2L12* (humanin‐like 12), derived from the mitochondrial gene *MT‐RNR2*, was markedly diminished in monocytes, T cells, SMCs, and ECs (Figure 2H and Figure S3), indicating altered mitochondrial function within the sarcoma microenvironment.

### 3.3 High heterogeneity of sarcomas cells and specifically transcriptional pattern

**FIGURE 3:**
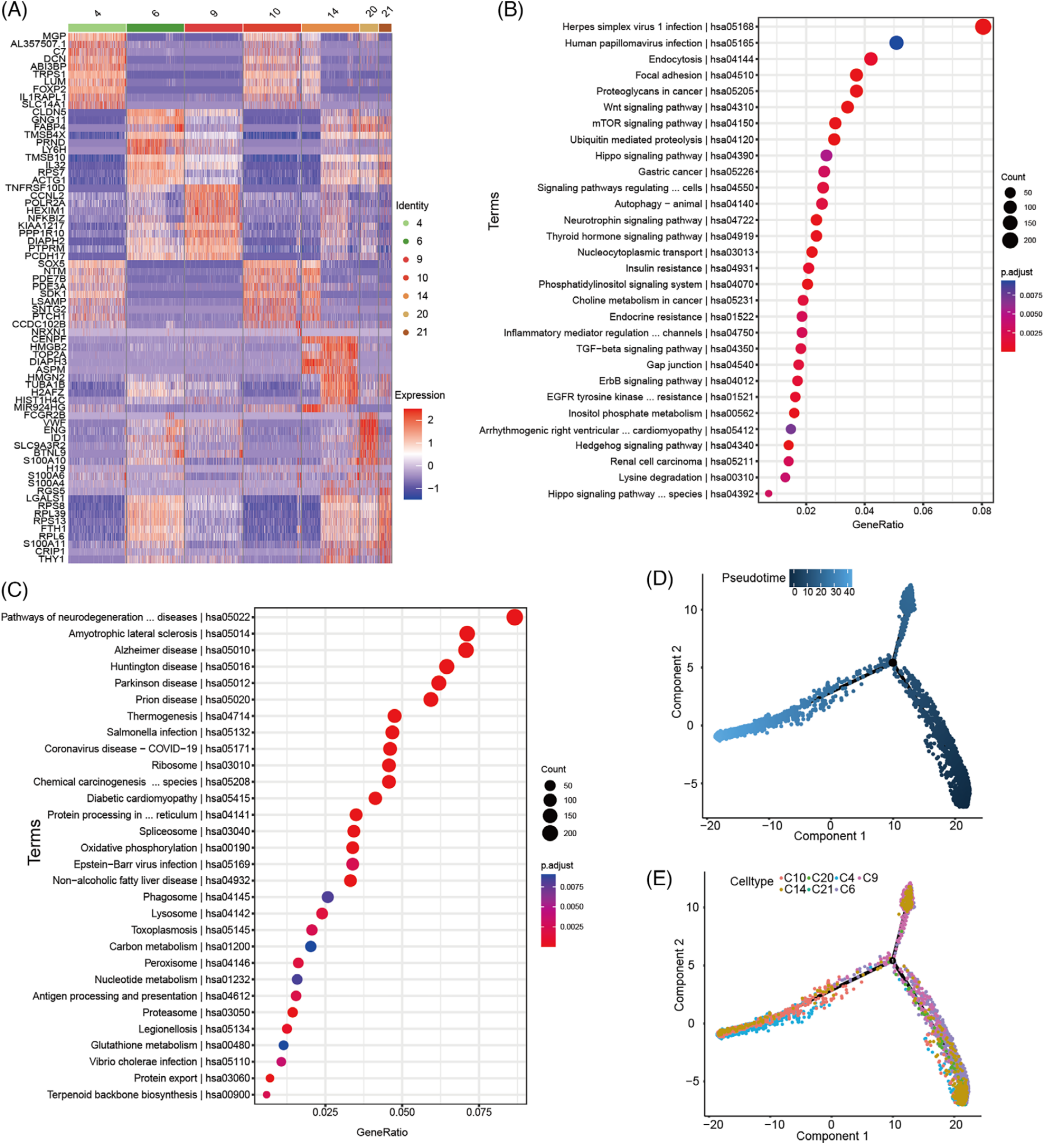
Characters of sarcoma cells. (A) Heatmap plot showing expression levels of selected marker genes for each cell type. (B, C) Kyoto Encyclopedia of Genes and Genomes (KEGG) enrichment analysis of genes with substantially up‐regulated in cluster 4 (B) and cluster 10 (C). (D, E) Trajectory reconstruction of sarcoma cells consisted of one pivot point coloured by pseudotime (D) or cell types (E).

To explore the characteristics of the sarcomas, we first calculated the CNV scores using InferCNV. As expected, clusters 4, 6, 9, 10, 14, 20 and 21 with high CNV scores from the mesothelial and sarcoma cell clusters were recognized as tumour cells (Figure S4A). Differential gene analysis of the subsets indicated that cluster 14 was a proliferating subset with high expression *CENPF*, *HMGB2* and *TOP2A*; clusters 4 and 10 were inflammation and urea metabolism pathway‐activated subsets with high expression *IL1RAPL1*, *C7*, *SLC14A1*, *PDE7B* and *PDE3A*; and clusters 6, 9, 20 and 21 were translation‐active subsets with high expression *RPS7*, *RPS8*, *PRL39*, *PRS13* and *RPL6* (Figure 3A). The KEGG pathway enrichment analysis also showed consistent results (Figure 3B,C and Figure S4B–F). Moreover, this proliferating cluster (cluster 14) occupied 3.88%, 8.96%, 7.84% and 9.42% of PCAS1‐4 samples respectively (Figure S4G). This cluster was also identified in sarcoma tissues (Figure S4H).

To understand the differential state of tumour cells, we also performed pseudotime analysis using Monocle2.^25^ As shown in Figure 3D,E, clusters 4 and 10 were relatively native subsets, clusters 6, 9 and 21 were relatively mature subsets and cluster 14 was relatively dispersed, indicating high heterogeneity. Pesudotime analysis of differential genes suggested that interferon‐stimulated genes (ISGs) such as *ISG15* and *IFI6* and inflammation genes such as *S100A10*, *S100A11*, *S100A16* and *ILF2* were activated in an early differentiation state. However, extracellular matrix *COL11A1*, *COL16A1* and NOTCH pathways such as *NOTCH2*, *NOTCH2NLC* and E3 ubiquitin‐protein ligases such as *FBXO42* (which specifically recognizes p53/TP53, promoting its ubiquitination *OTUD7B* and *UBE4B* were highly activated in later differentiation, indicating highly active protein update regulation and malignant potential (Figure S5A).^26^

**FIGURE 4:**
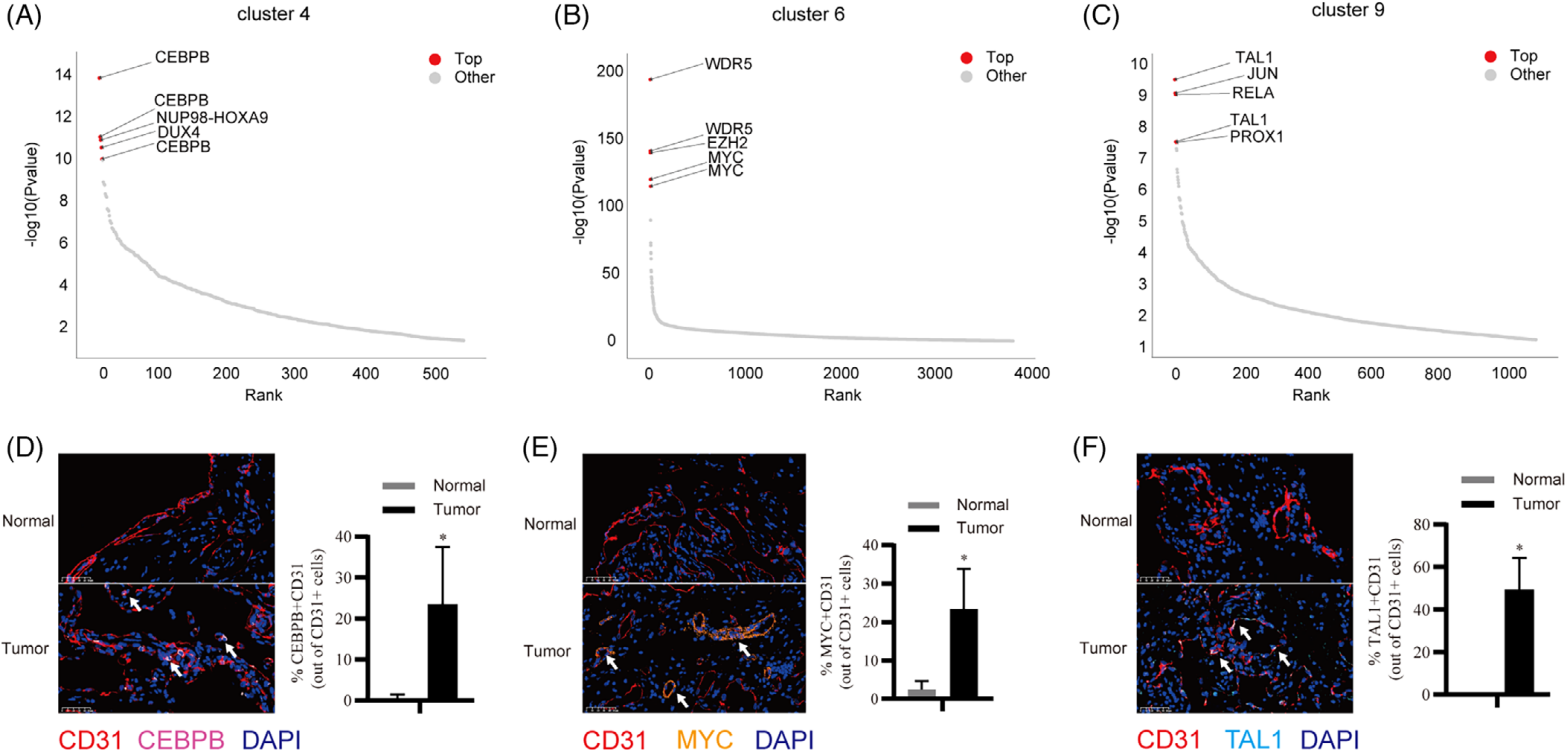
Characteristics of sarcoma cells. (A–C) Scatter plots showing the TOP five active transcription factors (TFs) in cluster 4 (A), cluster 6 (B) and cluster 9 (C) by SCENIC analysis. (D–F) Multiply immunohistochemistry (IHC) stain showing CEBPB+ sarcoma cells (D), MYC+ sarcoma cells (E) and TAL1+ sarcoma cells (F). The right panel is the qualified result.

Notably, SCENIC analysis revealed that there were mainly three kinds of TF expression with clusters 4 and 10 specifically expressing CEBPB and HOXA9; clusters 6 and 21 specifically expressing WDR5, EZH2 and MYC; cluster 9 specifically expressing TAL1 and RELA; and cluster 14 specifically expressing E2F4^27^ and FOXM1^28^ (Figure 4A–C and Figure S5B–E), suggesting that combined inhibitor usage may contribute to sarcoma therapy. Finally, mIHC staining was performed to confirm these results (Figure 4D–F).

### 3.4 Identification of gene module in sarcomas cells

**FIGURE 5:**
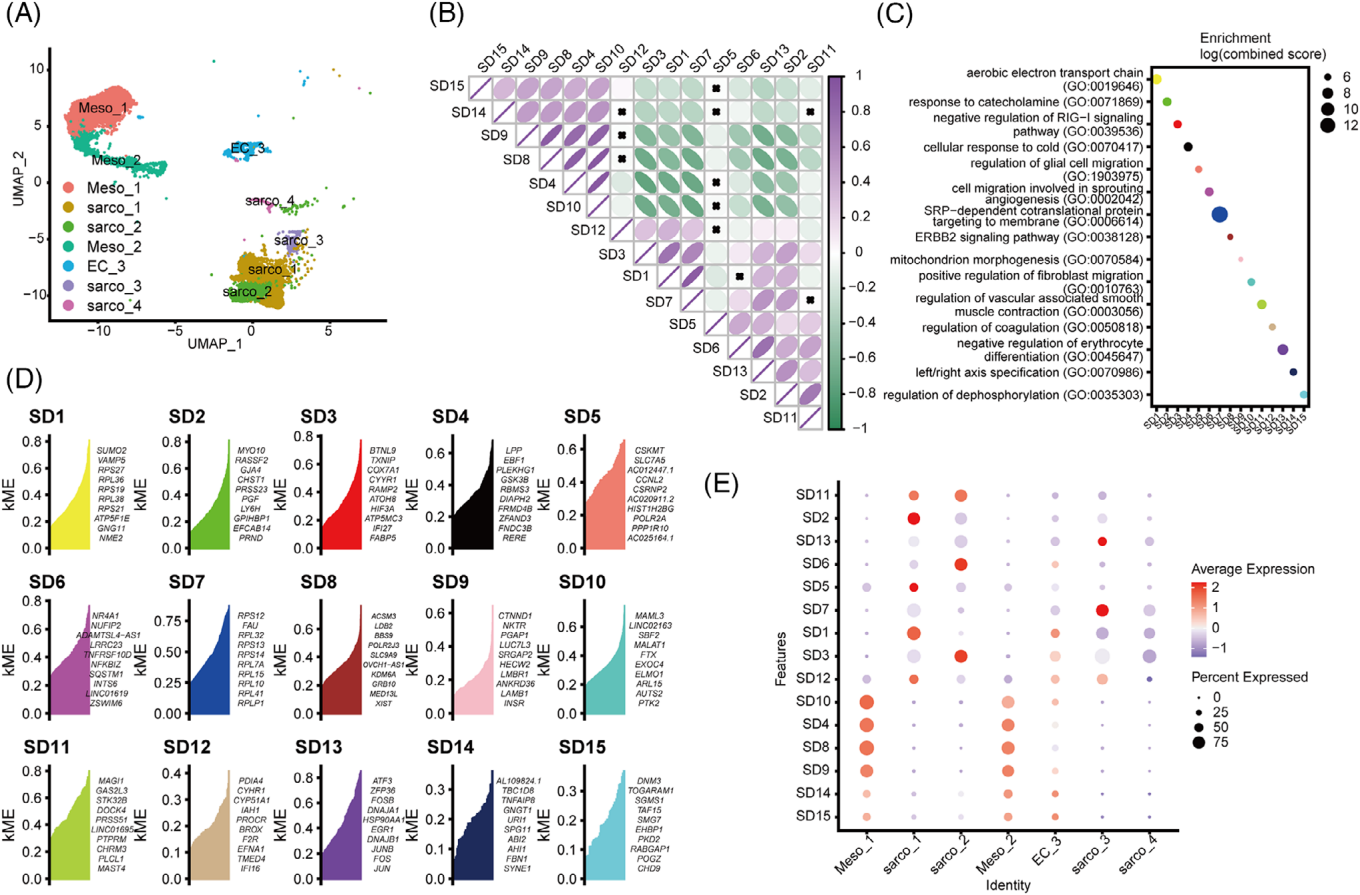
Gene module analysis of sarcoma cells. (A) Uniform manifold approximation and projection (UMAP) plot of sarcoma cells showing seven major cell types by manual annotation. (B) Correlation analysis of gene modules in sarcoma cells. (C) Dot plot showing selected Gene Ontology (GO) term enrichment results for each co‐expression module. (D) Bar plots coloured by module eigengenes (MEs) showing the TOP expression genes in each module. (E) Dot plots showing the expression frequency of each module in seven major cell types.

We conducted an hdWGCNA analysis to dissect the co‐expression networks within sarcoma cells (Figure 5A).^19^ After selecting the best soft‐power threshold (*n* = 9), we constructed a total of 15 co‐expression modules (SD, sarcoma modules) among sarcoma cells (Figure S6A,B). Among these modules, there were three kinds of modules: SD15, SD14, SD9, SD8, SD4 and SD10 had strong correlations representing one type of module, whereas SD3, SD1, SD7, SD6, SD13 and SD2 had strong correlations representing another type of module (Figure 5B). KEGG enrichment analysis of expression genes among the modules indicated that the SD7 module was enriched in translation‐related pathways and upregulated RPLs (Figure 5C,D). SD5, SD6, SD10 and SD11 were enriched in migration and angiogenesis, with high expression levels of CSKMT, NR4A1, MAML3, and DOCK4 (Figure 5C,D). Notably, SD3 was enriched in the negative regulation of RIG‐I signalling with high expression of BTNL9 and TXNIP, indicating endogenous anti‐immune activation and an anti‐antigen presentation module in sarcoma (Figure 5C,D).

To verify our hypothesis, we performed a niche analysis of sarcoma cells and T cells. Cell‐cell communication analysis showed that SD3 enriched sarcoma cell subtype sarco_2 (Figure 5E) had low expression of HLA‐DRA, HLA‐E, HLA‐F and HLA‐A, and high expression of ICAM1 and TGFB1 (Figure S6C,D). In contrast, sarco_1, with high HLA‐A and HLA‐E expression, was enriched in the SD1 and SD2 modules, which upregulate the aerobic electron transport chain and response to catecholamine pathways (Figure 5C,D). The above analysis suggests that complex gene modules control biological activities and are highly heterogeneous in sarcoma cells.

### 3.5 T cells exhaust differentiation in the cardiac angiosarcomas microenvironment

**FIGURE 6:**
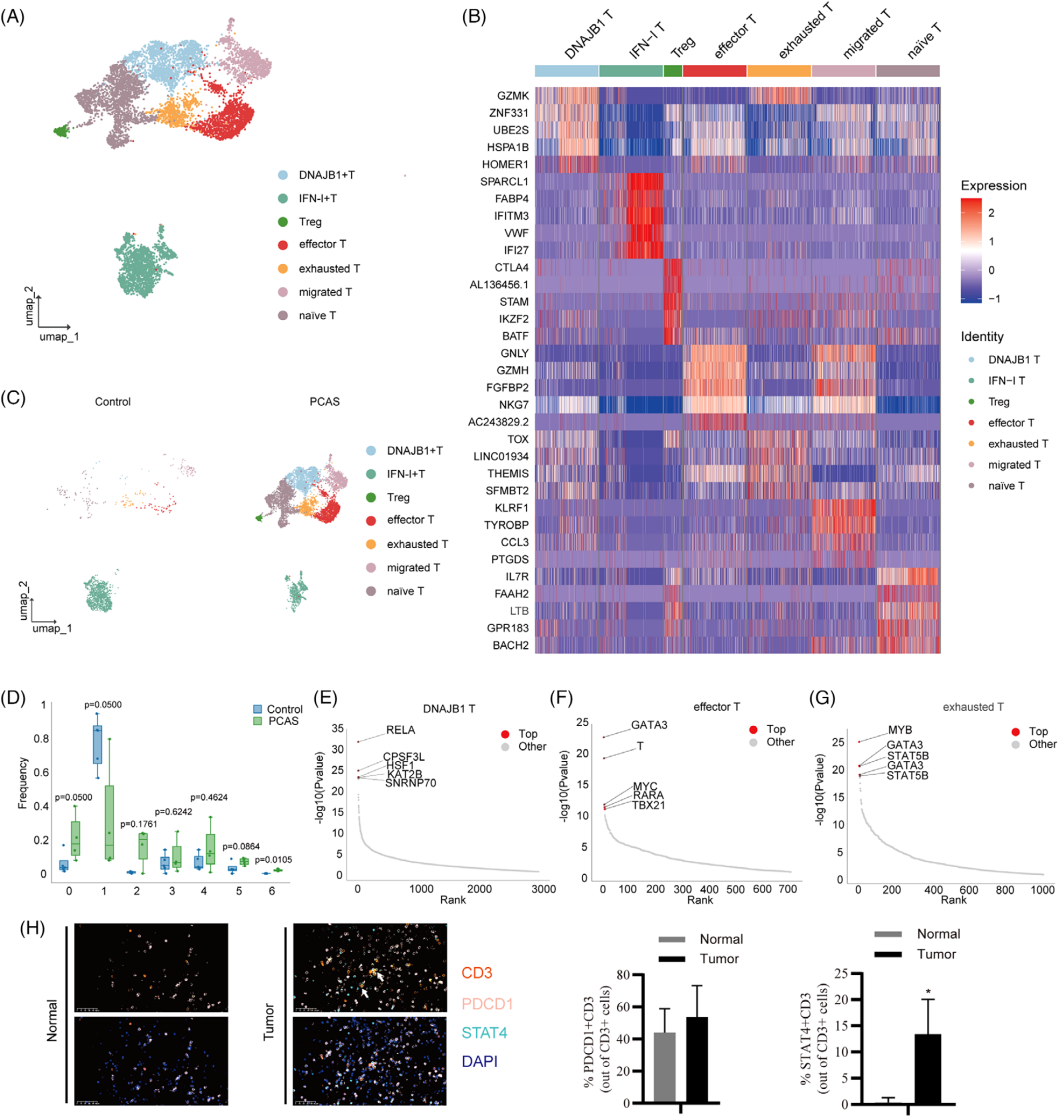
Landscape of T cells. (A) Uniform manifold approximation and projection (UMAP) plot of T cells showing seven major cell types by manual annotation. (B) Heatmap plot showing expression levels of selected marker genes for each cell type. (C) UMAP plot of T cells showing two types of samples. (D) Bar plot showing distributions of seven major cell types across two types of samples. (E–G) Scatter plots showing the TOP five active transcription factors (TFs) in DNAJB1_T (E), effector_T (F) and exhausted_T (G) by SCENIC analysis. (H) Multiply the immunohistochemistry (IHC) stain showing PDCD1+STAT4+ T cells. The right panel is the qualified result.

To systematically explore the state of the T subset in sarcoma tissues, we re‐clustered T cells into seven subsets using UMAP dimensionality reduction analysis (Figure 6A). Owing to the high level of basal Interferon (IFN)‐I expression in normal cardiac tissues,^29^ except for IFN‐I‐activated T (IFN‐I T subset), which was enriched in the normal group, other types of T subsets, including DNAJB1 T (DNA damage proteins enriched), Tregs, effector T, exhausted T, migrated T (*CCL3* and *TYROBP*) and naive T, were all enriched in the sarcoma group, indicating that a complex microenvironment induces the diversity of T cells (Figure 6B,C). Due to the highly differential microenvironments in different patients, specific enriched T subsets in each patient resulted in no obvious difference between the two groups in most T subsets, indicating that different sarcoma microenvironments induce particular T subset differentiation (Figure 6D).

Notably, the key exhaustion differentiation TF TOX was expressed in most T cell subsets, indicating that these different sarcoma microenvironments induce T cell dysfunction. Furthermore, we performed a SCENIC analysis of DNAJB1 T, effector T and exhausted T (TOP three abundant T subsets in the sarcoma group). RELA, HSF1 and KAT2B were activated in DNAJB1 T cells (Figure 6E). GATA3, TBX21 and MYC were activated in the effector T cells (Figure 6F). Immunofluorescence staining also showed that the JAK‐STAT1 pathway was activated (high STAT4+) in tumour‐infiltrating T cells, whereas the PDCD1+ ratio was not significantly different (Figure 6H, Figure S7J). KEGG pathways in these two T subsets were enriched in inflammation, Th17 differentiation, antigen processing and presentation, and GVHD, suggesting that these T subsets exhibit anti‐tumour effects (Figure S7A,B). MYB, GATA3 and STAT5B were also activated in exhausted T cells (Figure 6G). Furthermore, pathways including apoptosis and the TCR pathway were enriched in the exhausted T subset, indicating chronic persistent TCR activation in this type of T cell (Figure S7C). Finally, we compared the differential gene expression of T cell subsets between the tumour and normal tissues. As shown in Figure S7D–I, chemotactic factors (*CCL4* and *CCL5*), activated markers (*CD69* and *FOS*), ribosome genes (*RPLs* and *RPSs*), and antigen presentation factors (*HLAs*) were induced in tumour‐infiltrated T cell subsets, whereas mitochondrial genes (*MTRNR2L12* and *MT‐ND2*) were reduced, which is partly consistent with our previous data (Figure 2H and Figure S3).

### 3.6 Myeloid subsets analysis in the cardiac sarcoma microenvironment

**FIGURE 7:**
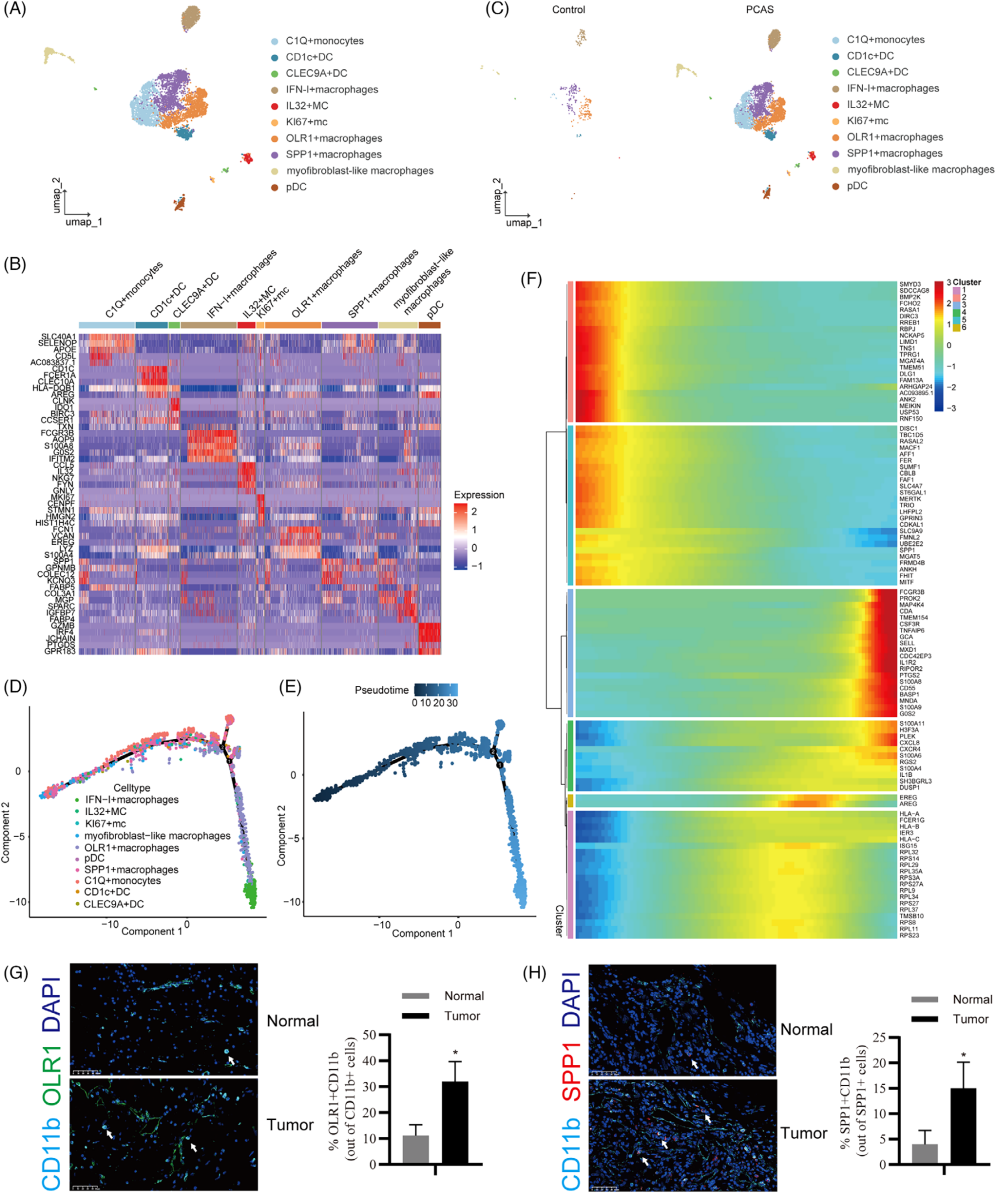
Landscape of myeloid cells. (A) Uniform manifold approximation and projection (UMAP) plot of myeloid cells showing 10 major cell types by manual annotation. (B) Heatmap plot showing expression levels of selected marker genes for each cell type. (C) UMAP plot of myeloid cells showing two types of samples. (D, E) Trajectory reconstruction of myeloid cells consisted of two pivot points coloured by cell types (D) or pseudotime (E). (F) BEAM Heatmap plot displaying the expression patterns of pseudotime‐specific genes of myeloid cells. (G, H) Multiply the immunohistochemistry (IHC) stain showing OLR1+ myeloid cells (G) and SPP1+ myeloid cells (H). The right panel is the qualified result.

As myeloid‐derived cells comprised most of the immune cells in the sarcoma group, we performed further cluster analysis on myeloid cell subsets using UMAP dimensionality reduction analysis (Figure 7A). Ten subsets were identified: C1Q+ macrophages, SPP1+ macrophages, IFN‐I + macrophages, OLR1+ macrophages, CD1c+ DC, CLEC9A+ DC and pDC (Figure 7B). As expected, both the cell types and counts increased in the PCAS group (Figure 7C). The main growth clusters were the SPP1+, OLR1+ and C1Q+ macrophages (Figure 7C). SPP1+ and OLR1 macrophages are suppressive immune cells that promote tumour progression.^30^, ^31^

Pseudotime analysis indicated that SPP1+ macrophages were a poor‐differentiated cell subset, while OLR1+macrophages were a high‐differentiation cell subset, which suggesting sarcomas microenvironment also induces matured macrophages phenotype transfer (Figure 7D,E). Importantly, all three types of macrophages were highly activated by proinflammatory TFs (SPI1, CEBPB and GATA3) (Figure S8A–C).

Differential gene expression analysis showed that RPLs, S100As, CDC42EP3, CXCL8 and FCGR3B were increased, suggesting that these subsets were translationally active (Figure 7F). However, many TFs and enzymes, including SMYD3, RREB1, RBPJ, BMP2K, CBLB, TMEM51 and RNF150 were downregulated. Interestingly, cytoskeleton proteins, including RASA1, RASAL2, NCKAP5, MACF1 and ARHGAP24, were reduced following differentiation, indicating that the sarcoma microenvironment induces resident‐like myeloid cell transfer (Figure 7F). Finally, using mIHC staining, we demonstrated that OLR1 + and SPP1 + macrophages were increased in angiosarcoma tissues (Figure 7G,H). Moreover, we calculated the infiltration scores of exhausted T cells (ex_T), OLR1 + macrophages (OLR1 + MC), and SPP1+macrophages (SPP1 + MC) in sarcomas treated with pazopanib (GSE156344). Unexpectedly, the infiltration scores of the three cell clusters did not differ significantly between the responsive and non‐responsive groups. However, these three clusters exhibited a decreasing trend in the responsive group (Figure S8D). The correlation analysis indicated a strong correlation between SPP1+ macrophages and ex‐T _T (Figure S8E). These analyses, based on public angiosarcoma data, partially identify our analysis of cardiac angiosarcomas.

## 4 DISCUSSION

Our study represents the first comprehensive analysis of PCAS at the transcriptomic level using single‐cell RNA sequencing. Unveiling a complex landscape, scRNA‐seq data revealed dense infiltration of diverse immune cell types, including macrophages, T cells in various states of exhaustion, and a range of poorly differentiated lymphocytes within the PCAS microenvironment. Notably, the abundant T cells in the TME exhibited a terminally exhausted differentiation profile, potentially elucidating the observed ineffectiveness of ICB in treatment.^5^ Additionally, our re‐cluster analysis highlighted a pronounced presence of immunosuppressive myeloid cells, particularly SPP1+ and OLR1+ macrophages, enriched within the TME. These cells may facilitate T‐cell exhaustion, suggesting a synergistic role in the resistance of malignancies to current therapeutic strategies. Additional in vivo and in vitro experiments are warranted to further validate these interactions and their implications, and provide a basis for future investigations and potential therapeutic targets.

Cell heterogeneity in sarcomas and the varied cellular composition of sarcoma tumours significantly complicate treatment and research efforts. DeMartino et al. explored this concept, focusing on rhabdomyosarcoma (RMS).^32^ Their study distinguishes between the cellular heterogeneity in fusion‐negative RMS, where cells show early myogenic differentiation traits, and fusion‐positive RMS, which mimics stages of skeletal muscle regeneration. Further research on undifferentiated pleomorphic sarcoma (UPS) using detailed single‐cell RNA sequencing has uncovered a complex cellular ecosystem within UPS tumours.^33^ This study identified multiple cellular populations, demonstrating substantial diversity and varying developmental trajectories within the tumours. Similar patterns of heterogeneity have been noted in other sarcoma types, including soft tissue sarcoma, osteosarcoma, and skeletal UPS (SUPS).^34^, ^35^, ^36^ Additionally, rare cardiac sarcomas share certain cellular features with more common sarcoma types. Our findings revealed multiple sarcoma cell clusters within these tumours, including both relatively native and mature subsets. This suggests that the microenvironment in PCAS significantly diversifies cell populations compared to that in normal cardiac tissue, with high levels of heterogeneity and distinct transcriptional profiles among different clusters.

The TME is a dynamic and complex area that surrounds and interacts with tumours within the body, comprising various immune cells, such as T cells, B cells, macrophages, and dendritic cells.^37^ These immune cells play dual roles: they may attack tumour cells to combat the tumour or paradoxically support tumour growth and aid in immune evasion.^37^ A recent study utilizing single‐cell RNA sequencing revealed a significant presence of FABP4+ macrophages in lung metastatic osteosarcoma lesions.^35^ These macrophages promote inflammation, which in the context of osteosarcoma, enhances the immune response, aiding tumour cell survival and invasion. Similarly, in soft‐tissue sarcomas, macrophages are dominant in the TME.^34^ The interaction between sarcoma cells and macrophages, mediated by MIF and the CD74 receptor, tends to push macrophages toward a tumour‐promoting state, thus supporting tumour growth. Our research also highlights a unique TME in cardiac sarcoma, marked by an increase in myeloid‐derived suppressor cells, specifically SPP1+ and OLR1+ macrophages, which are implicated in advanced tumour progression. Furthermore, we observed changes in the macrophage differentiation patterns, suggesting a transition towards more mature resident‐like phenotypes within the sarcoma microenvironment. This transition likely influences both sarcoma growth and immune response. Additionally, a decrease in the mitochondrial gene MT‐RNR2 product MTRNR2L12 in monocytes within the TME may contribute to these phenotypic changes.

T cells significantly affect cancer progression and the efficacy of immune‐based therapies within the TME.^38^ TME of RMS is notably immunosuppressive, marked by an abundance of regulatory T cells (Tregs) and the expression of immune checkpoint molecules such as TIGIT, which interact with NECTIN3 on cancer cells, potentially leading to T‐cell dysfunction.^32^ Single‐cell RNA sequencing has identified diverse T cell populations within the TME of SUPS, including exhausted CD8+ T cells and Tregs.^39^ These cells exhibit unique transcriptional profiles that contribute to tumour aggressiveness. The immunosuppressive character of the TME is further underscored by the high expression of checkpoint molecules, such as PD‐1, CTLA4 and TIGIT, on T cells.^39^ Additionally, transplant and primary sarcomas present contrasting immune landscapes. Transplant sarcomas are characterized by an enriched presence of activated CD8+ T cells and PD‐L1+ macrophages, whereas primary sarcomas generally show a less inflamed, more suppressive immune environment.^40^ In primary cardiac sarcomas, our findings similarly highlight an immunosuppressive TME. We observed various T cell subsets exhibiting exhaustion markers, indicative of a compromised immune response within the TME of cardiac angiosarcoma. The abundant expression of the TF TOX across these subsets suggests that diverse sarcoma environments could foster widespread T‐cell dysfunction, potentially affecting the success of immunotherapies. Additionally, the distinct gene expression profiles observed along the pseudo‐time trajectory, particularly those activating genes involved in inflammation and immune checkpoints, may contribute to resistance to chemotherapy and immune checkpoint inhibitors.

## 5 CONCLUSION

To our knowledge, this is the first study to document the transcriptomic characteristics of PCAS using single‐cell RNA sequencing. Our findings revealed that the immune microenvironment of PCAS is comparable to that of other solid tumours, characterized by a high prevalence of exhausted T cells and immunosuppressive myeloid cells. Notably, we observed significant downregulation of MTRNR2L12 expression in immune cells within the PCAS environment, suggesting a distinct impairment in mitochondrial function. This mitochondrial dysfunction may be a unique feature of the cardiac sarcoma microenvironment, underscoring the potential therapeutic targets specific to this malignancy.

## AUTHOR CONTRIBUTIONS

Jingyuan Huo: Data curation, software, investigation and writing of the original draft. Zhen Wang: Data curation, software, formal analysis and writing of the original draft. Wenting Zhao: Validation and visualization. Miao Chen: Data curation, software and formal analysis. Haoyang Li: Visualization. Fengpu He: Visualization. Xiao Tian: Data curation. Yaqi Ma: Data curation. Firyuza Husanova: Data curation. Liang Ma: Funding acquisition. Yiming Ni: Funding acquisition. Hongda Ding: Visualization. Weidong Li: Conceptualization, supervision and funding acquisition. Hongfei Xu: Conceptualization, supervision, methodology, funding acquisition and project administration.

## CONFLICT OF INTEREST STATEMENT

The authors declare no conflict of interest.

## ETHICS STATEMENT

Patients who donated samples provided signed informed consent, and the Ethics Committee of the First Affiliated Hospital of Zhejiang University Hospital approved the project. All the authors have reviewed and agreed to the submission of this manuscript. This manuscript has not been published or presented elsewhere.

## Supporting information



Supporting Information

## Data Availability

The raw sequence data reported in this paper have been deposited in the Genome Sequence Archive (Genomics, Proteomics & Bioinformatics 2021) at the National Genomics Data Center (Nucleic Acids Res 2022), China National Center for Bioinformation/Beijing Institute of Genomics, Chinese Academy of Sciences (GSA‐Human: HRA007415), and are publicly accessible at https://ngdc.cncb.ac.cn/gsa‐human. Additionally, the processed count matrix (deidentified), used for data transparency, has also been uploaded to the same database under the file name pbmc.RData. All other data generated and/or analyzed during this study are included in this published article.
