# Supplementary material for: Investigating intra‐tumoural heterogeneity and microenvironment diversity in primary cardiac angiosarcoma through single‐cell RNA sequencing
Source: Clin Transl Med. 2024 Dec 10;14(12):e70113. doi: 10.1002/ctm2.70113 (PMC11631565; doi:10.1002/ctm2.70113)
Supplement: Supplementary file 1 — Supporting Information [file CTM2-14-e70113-s001.docx]

**Supplemental material**

**Supplementary figures:**


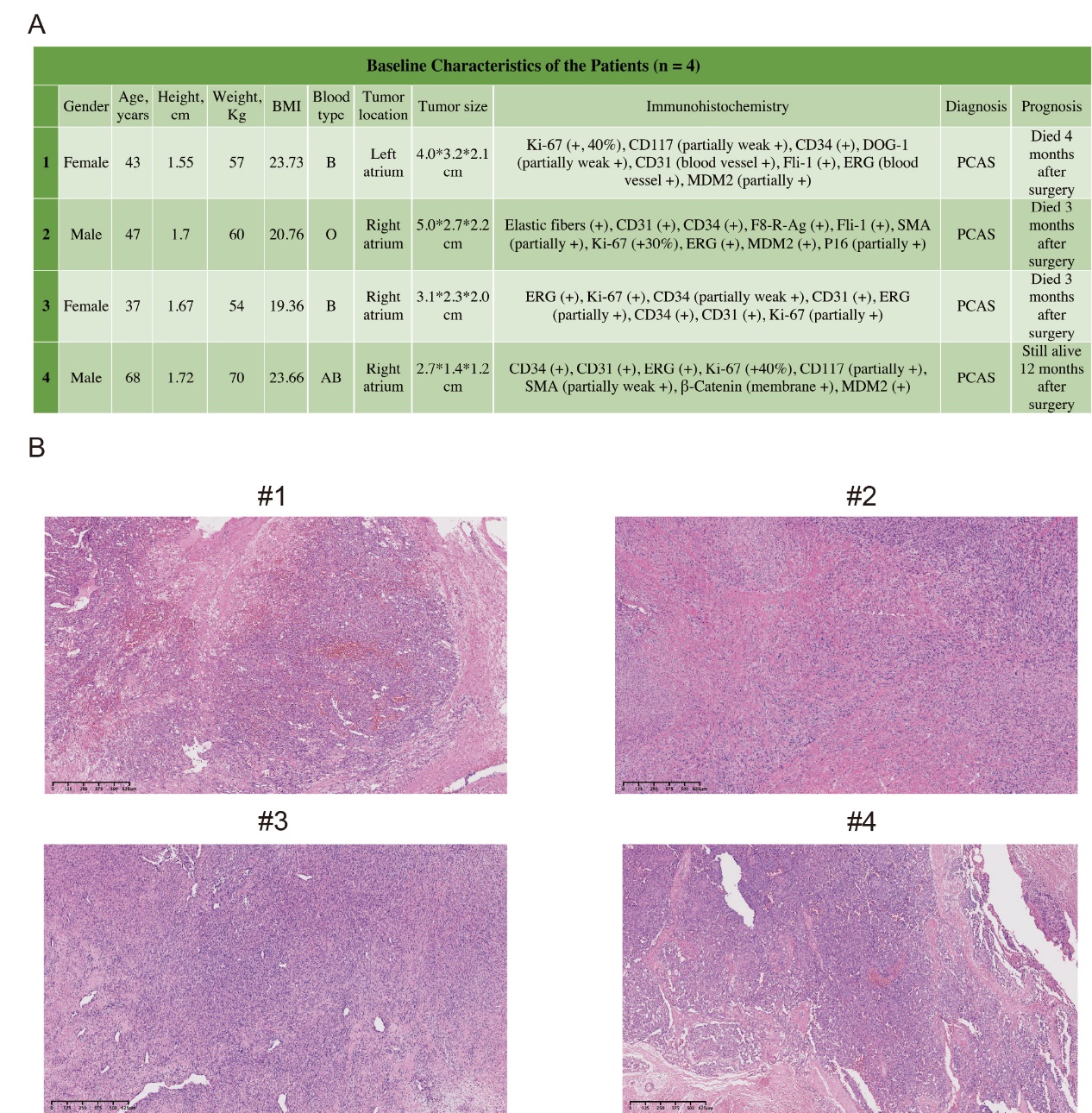


**Figure S1** Baseline characteristics of the PCAS patients.

A: Baseline characteristics of the 4 PCAS patients.

B: HE staining results of sarcomas from four patients.


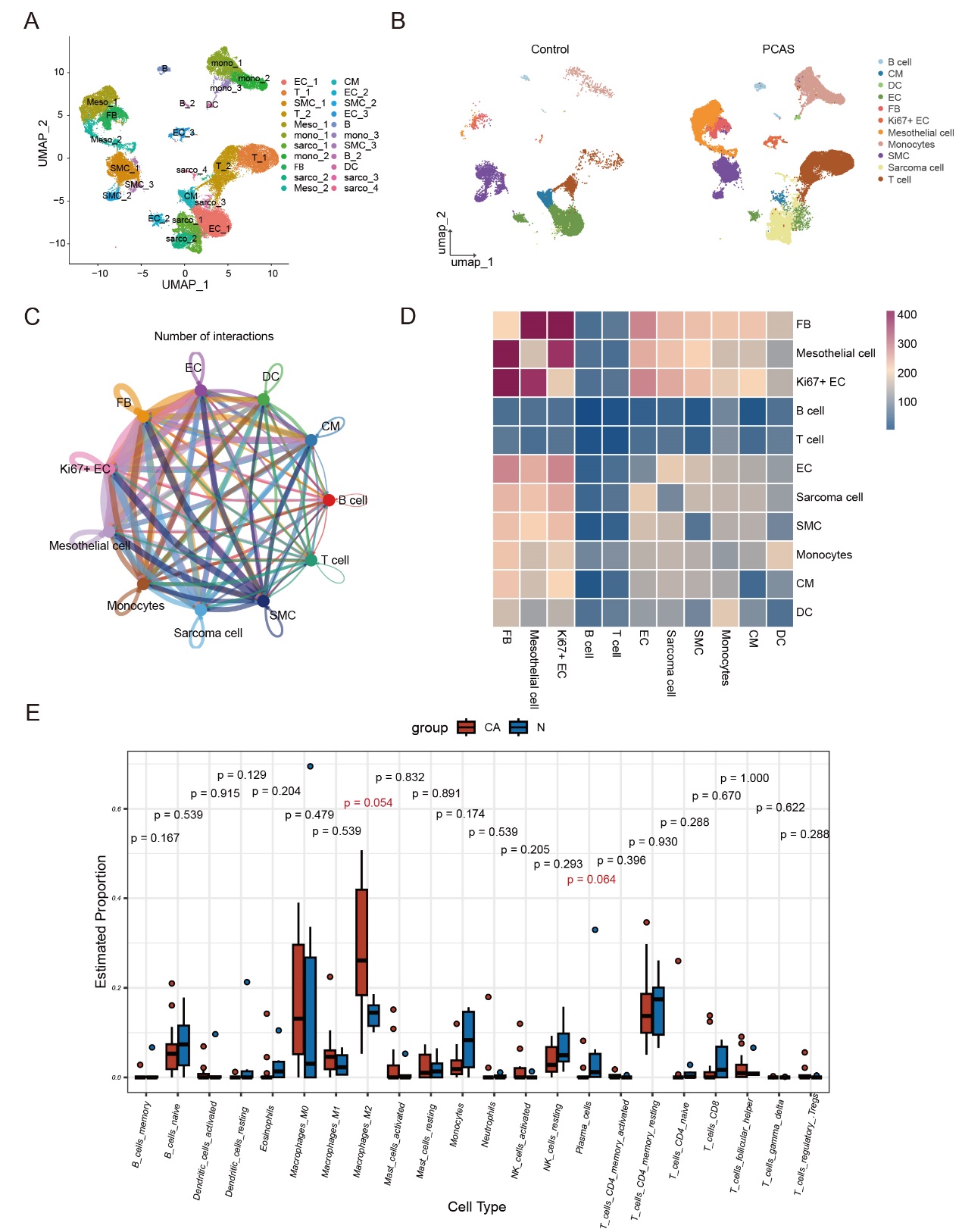


**Figure S2** Cellphone DB analysis among cell clusters in snRNA-seq. (A) Uniform Manifold Approximation and Projection (UMAP) plot of 10X genomics-based single cells showing 22 cell subsets by manual annotation. (B) UMAP plot of 10X genomics-based single cells showing 11 major cell types by manual annotation distribution in control and PCAS groups. (C) Chord diagrams showing the interactions strengthen among 11 major cell types in PCAS group. (D) Heatmap plot showing the interactions strengthen among 11 major cell types in PCAS group. (E) Heatmap plot showing the infiltration of immune cells in normal and tumor tissues from public database.


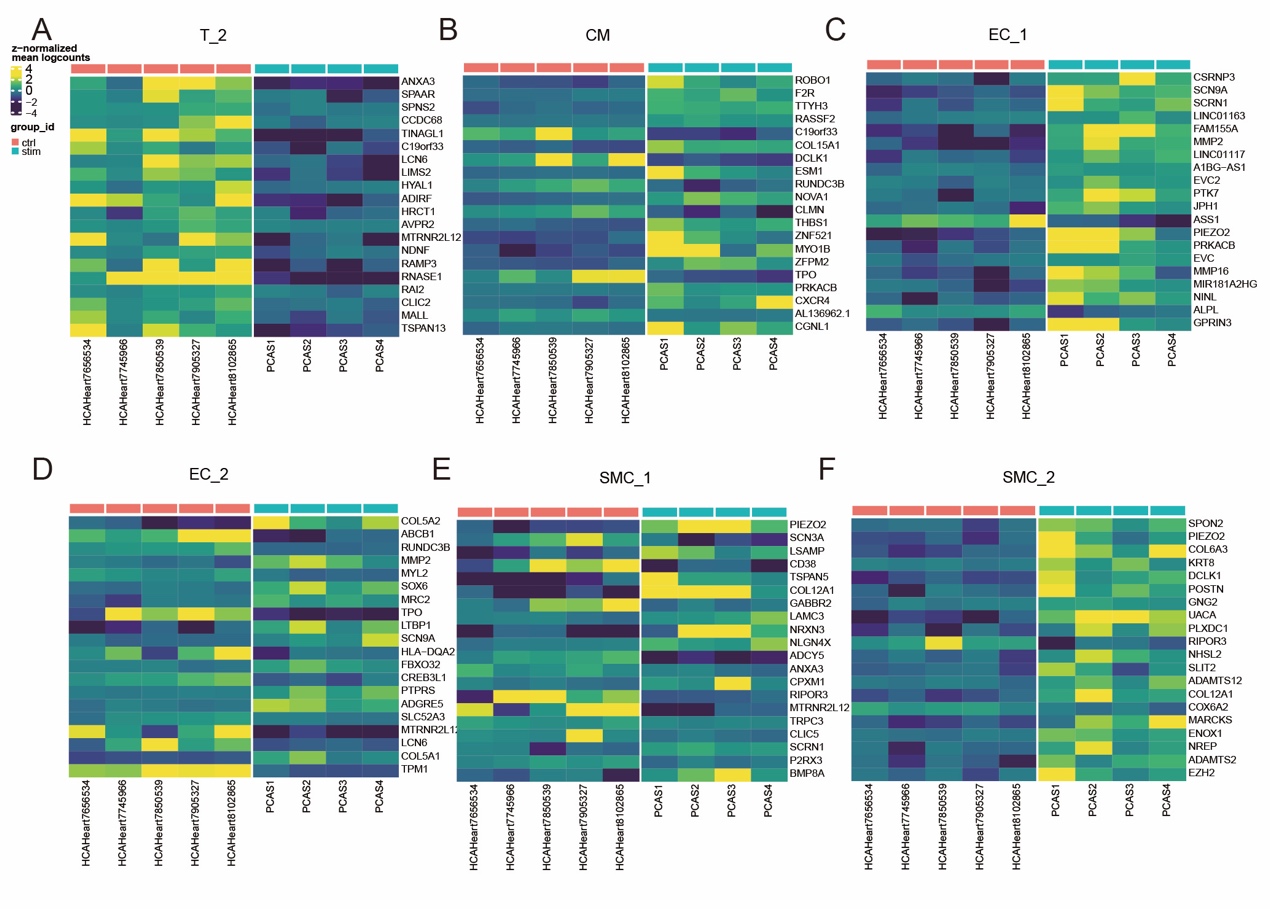


**Figure S3** Muscat analysis of non-tumor cells. (A-F) Heatmap of pseudobulk-level log-expression values normalized to the mean of vehicle samples; rows correspond to genes, columns to subpopulation-sample combinations. Included is the union of DS detections (FDR < 0.05 & log_2_FC > 1) across all subpopulations including T_2 (A), CM (B), EC_1 (C), EC_2 (D), SMC_1 (E) and SMC_2 (F).

**Figure S4** CNV analysis and KEGG enrichment analysis of tumor cells. (A) Boxplot showing the initial copy number variation (CNV) score of cell clusters in snRNA-seq. (B-F) Kyoto Encyclopedia of Genes and Genomes (KEGG) enrichment analysis of genes with substantially up-regulated in cluster 6 (B), cluster 9 (C), cluster 14 (D), cluster 20 (E) and cluster 21 (F). (G) Umapplot showing the distribution of cluster 14 in four sarcomas groups, and right panel is the qualified result. (H) Multiply immunohistochemistry (IHC) stain showing ki67+ sarcoma cells. Right panel is the qualified result.

**Figure S5** KEGG enrichment analysis of tumor cells. (A) BEAM Heatmap plot displaying the expression patterns of pseudotime-specific genes of tumor cells. (B-E) Scatter plots showing the TOP five active transcription factors (TFs) in cluster 10 (B), cluster 14 (C), cluster 20 (D) and cluster 21 (E).


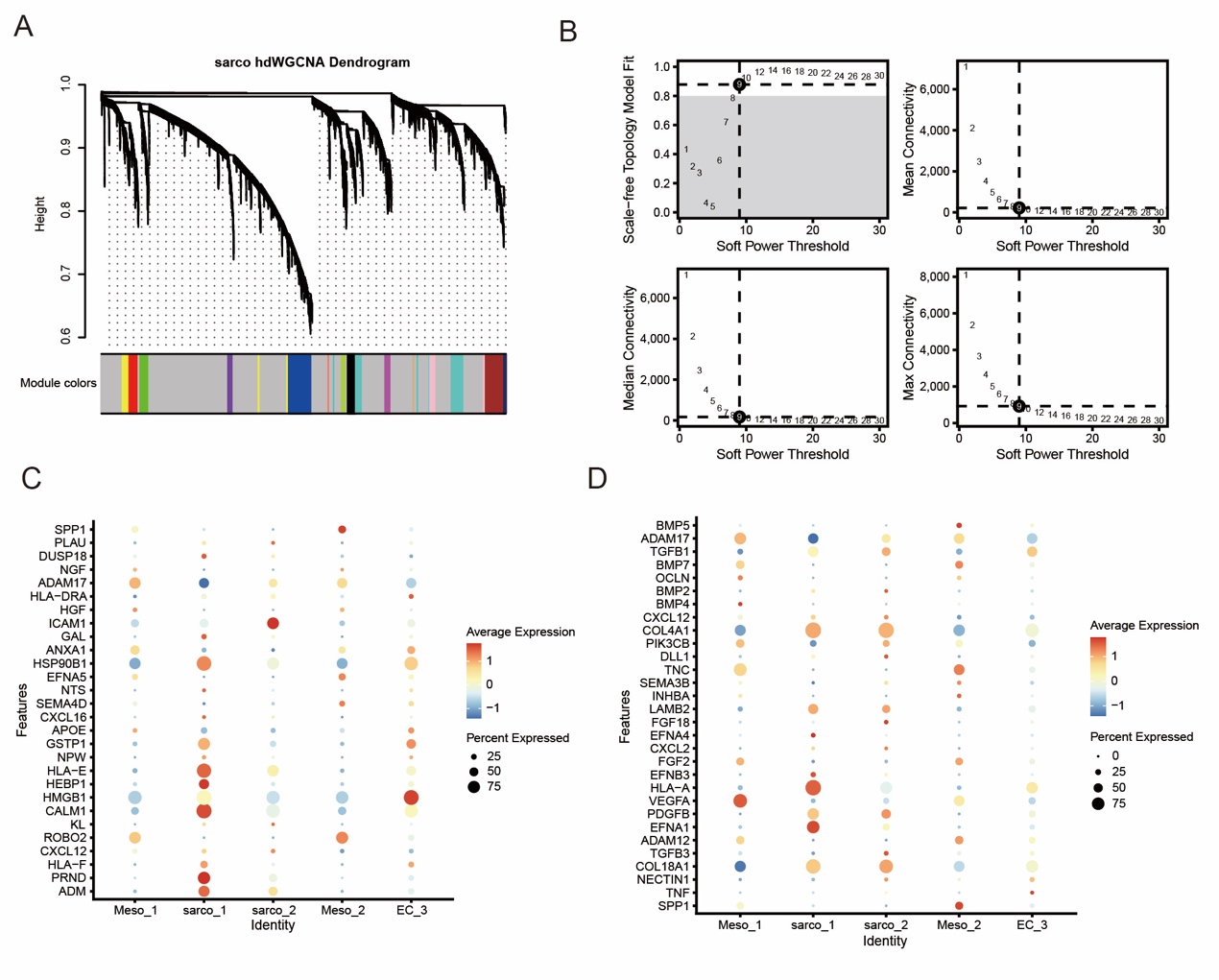


**Figure S6** Parameter setting in hd_WGCNA analysis and Ligand-receptor analysis by NicheNet. (A) Dendrogram plot showing the co-expression modules. The grey module should be ignored for all downstream analysis and interpretation. (B) Dot plots showing the parameter sweep in Scale Free Topology Model Fit and 9 was set as the lowest soft power threshold. (C) Dot plot showing the enriched LR-pairs in T_1 subset and tumor cells. (D) Dot plot showing the enriched LR-pairs in T_2 subset and tumor cells.

**Figure S7** KEGG enrichment analysis of T subsets. (A-C) Dot plots showing the enriched pathways in DNAJB1 T (A), effector T (B) and exhausted T (C). (D-I) Scatter plots showing the differential genes of DNAJB1+T (D), IFN-I+T (E), effector T(F), exhausted T (G), migrated T (H) and Treg (I) between normal and tumor groups. (J) Multiply immunohistochemistry (IHC) stain showing the specificity of αPD1 antibodies with αIgG used as negative control.

**Figure S8** SCENIC analysis of myeloid cells. (A-C) Scatter plots showing the TOP five active TFs in SPP1+ macrophage (A), OLR1+ macrophage (B) and C1Q+ macrophage (C) by SCENIC analysis. (D) Infiltration score calculation of exhausted T, OLR1+ MC and SPP1+ MC in therapy responsive and nonresponsive sarcomas. (E) Correlation analysis among exhausted T, OLR1+ MC and SPP1+ MC infiltration scores.

**Supplementary table:**

| **Supplemental Table 1 Seurat parameter** | | |
| --- | --- | --- |
|  | **Process** | **Filter criteria** |
| 1 | cell filtration | nGene >200； UMI < 99th percentile value；Proportion of mitochondrial gene < 25 (25%) |
| 2 | gene filtration | ncell>3 |
| 3 | Normalization parameters | LogNormalize,default |
| 4 | variable gene | selection.method = ""vst"", nfeatures = 2000 |
| 5 | ScaleData | features = all.genes, vars.to.regress = "percent.mt" |
| 6 | RunPCA | npcs = 50 |
| 7 | RunHarmony | group.by.vars= "sampleinf" |
| 8 | RunTSNE | reduction = "harmony", dims = 1:30 |
| 9 | RunUMAP | reduction = "harmony", dims = 1:30 |
| 10 | FindNeighbors | reduction = "harmony", dims = 1:30 |
| 11 | FindClusters | resolution = 0.6 |
| 12 | FindMarkers | immune.combined, ident.1 = gcase, ident.2 = gcontrol, verbose = FALSE |
